# The changing epidemiology of measles in an era of elimination: lessons from health-care-setting transmissions of measles during an outbreak in New South Wales, Australia, 2012

**DOI:** 10.5365/WPSAR.2016.7.1.010

**Published:** 2016-10-19

**Authors:** Alexis Pillsbury, May Chiew, Shopna Bag, Kirsty Hope, Sophie Norton, Stephen Conaty, Vicky Sheppeard, Peter McIntyre

**Affiliations:** a National Centre for Immunisation Research and Surveillance of Vaccine Preventable Diseases (NCIRS), The Children’s Hospital at Westmead and the University of Sydney, New South Wales.; b National Centre for Epidemiology and Population Health, The Australian National University, Australian Capital Territory.; c Western Sydney Local Health District, Parramatta, New South Wales.; d Sydney Local Health District, Camperdown, New South Wales.; e South Western Sydney Local Health District, Liverpool, New South Wales.; f Health Protection NSW, North Sydney, New South Wales.; g Discipline of Paediatrics and Child Health, University of Sydney, The Children’s Hospital at Westmead, Westmead, New South Wales.

## Abstract

**Introduction:**

In countries where measles is rare, health-care-setting transmissions remain problematic. Australia experienced its largest measles outbreak in 15 years in 2012 with 199 cases reported nationally; 170 cases occurred in the state of New South Wales (NSW) with symptom onset between 7 April and 29 November 2012.

**Methods:**

A descriptive study was conducted using measles case data obtained from metropolitan Sydney local health districts in NSW in 2012. Characteristics of measles source and secondary cases were described. Details of health-care presentations resulting and not resulting in measles transmission were also analysed.

**Results:**

There were 168 confirmed and two probable cases resulting in 405 documented health-care presentations. Thirty-four secondary cases acquired in health-care settings were identified, including 29 cases resulting from 14 source cases and 5 cases whose source could not be identified. Health-care-acquired cases accounted for 20% of all cases in this outbreak. Source cases were more likely to be of Pacific Islander descent (*P* = 0.009) and to have had more presentations before diagnosis (*P* = 0.012) compared to other cases. The percentage of presentations to emergency departments was higher for presentations that resulted in transmission compared to those that did not (71.4% and 37.6%, respectively, *P* = 0.028). There were no significant differences between transmission and non-transmission presentations with respect to presence of rash and infection control measures (*P* = 0.762 and *P* = 0.221, respectively), although the power to detect these differences was limited. Rash was reported at 66% of the presentations.

**Conclusion:**

Development of and adherence to protocols for the management of patients presenting to hospitals with fever and rash will minimize secondary transmission of measles.

## Introduction

Although Australia had been near measles elimination since 2005 (*1*) and was declared to have officially eliminated measles in 2014, (*2*) Australia experienced its largest measles outbreak in 15 years in 2012 with a total of 199 cases reported nationally. The number of cases has remained high since then with 340 confirmed cases (14.39 per 1 000 000 population) in 2014. (*3*) There were 170 cases in the state of New South Wales (NSW, Australia’s most populous state) in the 2012 outbreak with the index case having symptom onset on 7 April and the last case on 29 November, among whom 168 were confirmed. (*4*) Western Sydney, where the majority of outbreak cases resided, is culturally diverse. Over a third of its two million population were born overseas, and it also includes a very large urban population of Aboriginal and Torres Strait Islander people. (*5*-*7*)

In countries where measles is rare, transmissions in health-care facilities have been important in amplifying outbreaks (*8*, *9*) and challenging retention of measles elimination status. Although numerous measles outbreak reports have been published describing health-care transmissions, (*10*-*12*) many lack details of case demographics and transmission characteristics that are crucial for improving control and response guidelines for post-elimination settings.

The 2010 NSW Public Health Act requires all measles patients to be notified to local public health units by doctors and laboratories. (*13*) Health-care-setting transmissions of measles in NSW were well documented in the 2012 Australian outbreak. This study describes key characteristics of health-care transmissions in this NSW outbreak, including the clinical setting and timing of presentations, the ability of clinicians to efficiently identify a probable measles case and the stage of illness of presenting cases.

## Methods

A descriptive routine-databased study was conducted to compare characteristics of the measles cases who met the definition of a source case and cases who presented to a health-care facility and did not transmit illness. Characteristics of individual presentations to health-care facilities were also described.

### Data source

Case series data describing both confirmed and probable measles cases, as defined by Australian national guidelines, (*14*) with symptom onset between 7 April and 29 November 2012 were obtained from metropolitan Sydney local health districts (LHDs) that conducted case interviews in NSW. Collected data included age, sex, ethnicity and/or country of origin, second language, number of health-care presentations before diagnosis and vaccination status. Vaccination status was categorized as fully vaccinated, partially vaccinated, not vaccinated, too young to be vaccinated or unknown, according to the data recorded by the public health units in the NSW Notifiable Conditions Information Management System (NCIMS). For most cases, their vaccination status relied upon self- or parental-recall. Where complete, details in the vaccination validation field in NCIMS that documented written evidence of vaccination history, such as Australian Childhood Immunization Register (ACIR) or health records, were used to assist categorization of vaccination status. Data regarding time of arrival and discharge from health-care facilities were obtained from emergency department (ED) records or from general practice (GP) clinic records where available.

### Definitions of study parameters

A health-care facility was defined as any premise that delivers health-care services including hospital EDs, inpatient wards and GP clinics. A presentation was defined as a case who sought care at a health-care facility. A transmission event in a health-care setting was defined by the discovery of a measles case arising 7–18 days after a visit to the same health-care setting at approximately the same time as an infectious case. A (known) source case was defined as a measles-infected individual who transmitted the disease to another previously uninfected individual. A secondary case was defined as a previously uninfected individual who was infected by a source case in a health-care facility. If more than one case had symptom onset at the same time and presented in the same health facility on the same day with likely overlap in time and location, these cases were also considered as secondary cases even though the source cases could not be determined. Secondary cases were only classified as having been infected in a health-care setting if there was no other more likely source of transmission (e.g. household).

### Data analysis

Demographic details of the measles cases in the outbreak were summarized. Characteristics of the measles source cases and cases who presented to a health-care facility and did not transmit illness were compared. Characteristics of individual health-care presentations were described to compare health-care presentations that led to transmission events and those that did not.

Overlap times in health-care facilities for presentations that resulted in transmission with the presentation times of their subsequent secondary cases were estimated by calculating the difference in minutes between recorded arrival and discharge times. χ^2^ tests were conducted to compare categorical variables, including age group distribution, sex and vaccination status between those cases or presentations that resulted in transmission events and those that did not. A *p*-value of less than 0.05 was considered statistically significant. All analyses were done using Stata version 12 (StataCorp LP, College Station, TX, USA). When conducting χ^2^ tests comparing presentations, we used survey commands to adjust for clustering of observations within patients. A Mann–Whitney test was used for all analyses comparing medians of numbers of presentations before diagnosis between cases who transmitted and those who did not. For medians of time spent in a health-care setting and day of illness when presenting to health care, no statistical test was conducted to compare presentations that led to transmissions to those that did not due to the complexity of clustering effect.

### Ethics

Ethics approval was not required for this study as it was part of the public health outbreak response conducted under the NSW Public Health Act. (*13*)

## Results

### Characteristics of the measles cases

**Fig. 1:**
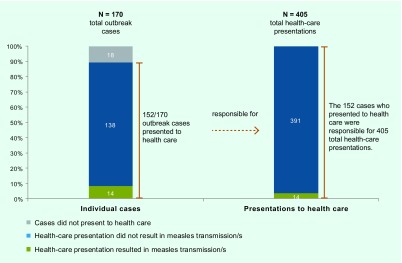
Overview of the total number of measles cases and presentations analysed, NSW, Australia, 2012

From 7 April to 29 November 2012 in NSW, there were 168 confirmed and two probable measles cases. (*14*) Of these 170 cases, 152 presented a total of 405 times to various health-care settings during the outbreak (**Fig. 1**). Of the total presented cases, 43 (28.3%) were aged 10–19 years and 80 (52.6%) were male. Thirty-four (22.4%) were of Pacific Islander descent. Twenty-six (17.1%), were reported as fully vaccinated and eight (5.3%) as partially vaccinated (**Table 1**). Only seven (20.6%) of those reported to be fully or partially vaccinated were noted in the NCIMS database as having documented evidence of their vaccination status including written health record or inclusion in the ACIR. Fourteen (9.2%) cases met the definition of source case and were linked to 29 health-care-acquired secondary cases; two unknown source cases were linked to a further five health-care-acquired cases, resulting in a total of 34 identified secondary cases. This represents 20.2% of all laboratory confirmed cases.

**Table 1:** Demographics of total measles outbreak cases who presented to health-care facilities and total health-care-acquired measles cases, NSW, Australia, 2012

-	Total outbreak cases who presented to a health-care facility* (*n* = 152)		**Total health-care-acquired** **(secondary) cases** **(*n* = 34)**	
	**Number**	**Proportion**	**Number**	**Proportion**
Age group				
< 1 year	36	23.7%	12	35.3%
1–9 years	23	15.1%	7	20.6%
10–19 years	43	28.3%	3	8.8%
20–59 years	49	32.2%	12	35.3%
≥ 60 years	1	0.7%	-	-
Sex				
Male	80	52.6%	21	61.8%
Female	72	47.4%	13	38.2%
Vaccination status				
Fully vaccinated	26	17.1%	8	23.5%
Partially vaccinated	8	5.3%	2	5.9%
Ineligible (aged < 12 months)	38^†^	25.0%	10	29.4%
Not vaccinated	52	34.2%	9	26.5%
Unknown	28	18.4%	5	14.7%
Pacific Islander status				
Pacific Islander	34	22.4%	3	8.8%

* Of 170 total cases, 152 presented a total of 405 times to various health-care settings during the outbreak.

† These included two cases who had just reached 12 months of age at symptom onset and health-care staff decided they were “too young to be vaccinated.”

### Health-care-acquired (secondary) cases

The median age of the health-care-acquired cases (*n* = 34) was 5.5 years (range: 0–37 years). Ten cases (29.4%) were infants too young to be vaccinated, nine (26.5%) were unvaccinated, two (5.9%) were partially vaccinated and eight (23.5%) were fully vaccinated. The vaccination status for the remaining five cases was unknown (**Table 1**). One case (2.9%) was a health-care worker. Three secondary cases (8.8%) were documented as Pacific Islanders (**Table 1**).

### Comparison of source cases and cases who did not transmit measles

The median age of the 14 known source cases (15.5 years; range: 0–38 years) was not statistically different from the median age of those 138 cases who presented to a health-care facility but did not transmit infection (14.5 years; range: 0–61 years). Similar proportions in both groups were unvaccinated (35.7% versus 34.1%) or too young to be vaccinated (21.4% versus 25.4) (**Table 2**). Though **Table 2** indicates that 26 total cases were fully vaccinated, only three of these cases had their vaccination status validated by a written health record or inclusion in the ACIR; all of these were non-transmitters. A significantly higher percentage of source cases were of Pacific Islander decent compared to cases that did not lead to health-care-acquired transmission (50.0% versus 19.6%, *P* = 0.009).

**Table 2:** Demographics of measles cases presenting to health-care facilities that resulted in transmission (source cases) versus no transmission, NSW, Australia, 2012

**-**	Total number of cases presenting to a health-care facility (*n* = 152^†^)				P-value
	Transmission (*n* = 14)		No transmission (*n* = 138)		
	Number	Proportion	Number	Proportion	
Age group					
< 1 year	3	21.4%	35	25.4%	0.290
1–9 years	2	14.3%	19	13.8%	
10–19 years	5	35.7%	38	27.5%	
20–29 years	3	21.4%	15	10.9%	
30–39 years	1	7.1%	25	18.1%	
≥ 40 years	-	-	6	4.4%	
**Sex**					
Male	8	57.1%	72	52.2%	0.723
Female	6	42.9%	66	47.8%	
**English as a second language**					
Yes	1	7.1%	3	2.2%	0.327
No	11	78.6%	101	73.2%	
Unknown^§^	2	14.3%	34	24.6%	
**Vaccination status**					
Fully vaccinated	3	21.4%	23	16.7%	0.973
Partially vaccinated	1	7.1%	7	5.1%	
Ineligible (aged < 12 months)	3	21.4%	35	25.4%	
Not vaccinated	5	35.7%	47	34.1%	
Unknown^§^	2	14.3%	26	18.8%	
**Pacific Islander**					
Yes	7	50.0%	27	19.6%	0.009*
No	7	50.0%	111	80.4%	
**Number of presentations before diagnosis**					
-	Median: 3.5 presentations (range: 2–5 presentations)		Median: 2.0 presentations (range: 1–7 presentations)		0.012*

† Of 170 total cases, 152 presented a total of 405 times to various health-care settings during the outbreak.

* Indicates statistical significance.

§ Unknowns not included in the χ2 analysis.

All cases who resulted in transmission presented on more than one occasion before successfully receiving a diagnosis (range: 2–5 presentations). The median number of presentations among cases that resulted in transmission was statistically higher than those who did not (3.5 presentations versus 2.0 presentations, *P* = 0.012).

### Presentations

Of the 405 presentations, 14 (3.5%) resulted in transmission. Two hundred and sixty-nine presentations (67.8%) included a rash at presentation and 377 (96.2%) included a cough. A total of 104 presentations occurred on weekends (26.1%). There were 157 (39.2%) presentations to an ED and 195 (48.6%) to a GP. In 148 (39.6%) presentations, infection control measures were reported by physicians, including giving patients masks, locating them in a single room and others.

#### Presentations resulting in transmissions versus those that did not

##### Presentation setting

In presentations that led to transmission, ED visits were significantly over-represented (71.4% versus 37.6%) and GP visits significantly underrepresented (14.3% versus 49.4%) compared with presentations not resulting in transmission (*P* = 0.028; **Table 3**).

**Table 3:** Information by health-care presentations that resulted in measles transmission versus no transmission, NSW, Australia, 2012

** -**	Total number of health-care presentations (n = 405)					P-value
	Transmission (n = 14)			No transmission (n = 391)		
	Number		Proportion	Number	Proportion	
**Rash at presentation**						
Yes	10		71.4%	259	66.2%	0.762
No	4		28.6%	124	31.7%	
Unknown^§^	-		-	8	2.1%	
Day of week						
Weekday	8		57.1%	287	73.4%	0.141
Weekend	6		42.9%	98	25.1%	
Unknown^§^	-		-	6	1.5%	
**Cough at presentation**						
Yes	14		100.0%	363	92.8%	0.565
No	-		-	15	3.8%	
Unknown^§^	-		-	13	3.3%	
**Health-care setting**						
Emergency department	10		71.4%	147	37.6%	0.028*
General practice	2		14.3%	193	49.4%	
Hospital ward	2		14.3%	47	12.0%	
Unknown^§^	-		-	4	1.0%	
**Infection control measures**						
Yes		3	21.4%	145	37.1%	0.221
No		10	71.4%	216	55.2%	
Unknown^§^		1	7.1%	30	7.7%	
**Median time spent in a health-care setting (hours)^||^**						
-		15.0^ᵻ^ (range: 2.3–2212.0 hours)		4.9^±^ (range: 0–10080.8 hours)		-
Day of illness when presenting to health-care^||^						
-		Median: 3.5 days (range: 1–8 days)		Median: 3.0 days (range: 0–15 days)^‡^		-

§ Unknowns not included in the χ2 analysis.

* Indicates statistical significance.

|| Statistical tests not conducted for continuous variables.

ᵻ Time unknown for one presentation.

± Time unknown for 153 presentations.

‡ Day of illness unknown for 10 presentations.

##### Presentation time

The median time of presentations which resulted in transmission was longer than those presentations which did not result in transmission (15.0 hours versus 4.9 hours). While 42.9% of presentations that resulted in transmission occurred on a weekend, 25.1% of those that did not result in transmission occurred on a weekend, although the difference was not significant (*P* = 0.141). Of the presentations that resulted in transmission, those on weekends had a median time of 33.1 hours (range: 6.6–2212.0 hours) while those on weekdays had a median time of 4.6 hours (range: 2.3–108.8 hours) (data not shown).

##### Stage of illness of presenting case

The median day of illness for presentations resulting in transmission was 3.5 (range: 1–8 days) compared with 3.0 days (range: 0–15 days) for those presentations which did not (**Table 3**). Rash was reported at 71.4% presentations that resulted in transmission, compared to 66.2% of those that did not (*P* = 0.762). On average, 2.3 secondary cases resulted from presentations with rash compared with 1.5 secondary cases for presentations without rash (data not shown).

##### Overlap time for secondary infections

The overlap time between presentations that resulted in transmission and their subsequent secondary cases was estimated for 10 of the 12 transmission events in hospital (ED and wards) for which the source cases could be identified; the median was 4.4 hours (range: 59 minutes – 35.5 hours). All secondary cases were present at the same time as the case who was the source of their infection. For one of the two transmission events for which a source case could not be identified, the four resultant secondary cases each overlapped in time. For the other transmission event with no identifiable source case, the secondary case was present in the ED at the same time as two source cases so we could not ascertain which source case was responsible for the infection. Overlap times for presentations that resulted in transmission in GP clinics could not be estimated because arrival and departure times of patients were not typically recorded; however, one of the three secondary cases acquired in a GP clinic reported that a measles case was known to be present during a concurrent visit.

## Discussion

In countries where measles is rare and most clinicians have not experienced a case first hand, (*9*, *15*) measles may go undiagnosed and outbreaks may result. A recent review found that up to 50% of cases in developed countries, particularly where measles elimination was established, had been acquired in a health-care setting. (*16*) In the 2012 NSW outbreak, we found approximately 20% of cases were infected in health-care facilities.

The reasons for the predominance of health-care-setting transmissions are obvious. Cases are contagious from four days before to four days after the rash appears. (*14*) At first presentation, few cases are suspected of having measles because clinically distinguishing it from other viral systemic illnesses is problematic. (*17*) A patient in the early stages of measles may present with a combination of non-differential symptoms, including fever and perhaps only one of the following: cough, coryza and conjunctivitis. Differential diagnoses include influenza and other common respiratory viral infections and allergic rhinitis. Even with the characteristic maculopapular rash, a measles diagnosis may be overlooked because of the disease’s rareness and similarities to adeno- and enteroviral infection, other exanthema of childhood and drug allergy. (*9*, *18*) In this outbreak, unable to obtain a successful diagnosis on first presentation, most source cases presented multiple times. Cases who transmitted measles were more likely to have multiple presentations compared with those who did not transmit the virus and were more likely to be of Pacific Islander descent.

In ED settings where ill individuals congregate in close proximity, often for long periods of time, transmission is particularly problematic. In this outbreak, presentations that resulted in measles transmission were significantly more likely to be in an ED. This could be influenced by the fact that particularly vulnerable individuals such as young infants and the immunocompromised may be more likely to present to an ED as compared to a GP for their illness. Our data demonstrated that transmissions were also more likely to have occurred among presentations that lasted longer.

In addition to documenting the lengthiness of presentation times, our data also revealed that all transmissions for which a source case could be identified occurred during a direct overlap in time between the presentation of source and secondary cases, echoing similar findings from a 2011 NSW outbreak. (*19*) This evidence influenced the Communicable Diseases Network of Australia to amend its Series of National Guidelines for measles control. It is now recommended that contact tracing only be conducted for contacts present in a location for up to 30 minutes after the source case is known to have departed, rather than for two hours as was previously advised. (*14*) As previous Australian research estimated the expenses associated with managing 75 contacts of one measles case in a 2011 outbreak as 2433 Australian dollars, (*20*) reduction in contact-tracing expenditure in future outbreaks could be substantial. (*19*)

Our results identified that even during the 2012 outbreak’s peak, when multiple public health alerts had been disseminated to health-care facilities, several measles cases despite presenting with rash were not suspected of having measles at the first presentation. The need for clinicians to maintain a high suspicion of measles during times of outbreaks cannot be overemphasized. (*21*) In the future, more innovative approaches may be required to improve such control efforts, including establishing alerts that are triggered when ‘fever’ and ‘rash’ are entered into electronic medical records. Such measures, however, have yet to be evaluated. (*22*, *23*) In addition to improving timely recognition and diagnosis of measles cases, control of the 2012 outbreak could have benefited from consistent and standardized infection control measures. (*14*) Although several source cases were recorded as having been subjected to infection control measures, efforts were ineffective or enacted too late to prevent transmission. Infection control was documented to have differed not only between hospitals but also within hospitals. Admittedly, measures may not have been rigorously documented in this outbreak.

As is common with studies based on retrospectively collected data, data completeness and quality presented significant limitations to the interpretations we could draw from our analysis and to the analyses we were able to conduct. Accuracy of routine clinical documentation limited our ability to compare transmission risk between source and non-source cases. While hospital data systems allow accurate recording of measures such as arrival and departure time, given the extremely long periods of time that some patients were determined to have been present in hospital, it is possible that even these measures are not always accurate. Other infection control measures are not uniformly reported and could range from actions that haven’t been proved to be effective such as giving a patient a mask to locating them in a single room with negative pressure ventilation. Vaccination status is rarely confirmed against medical records by assessing clinicians, and there is known underreporting of vaccination to the ACIR, limiting public health units’ ability to confirm vaccination histories; vaccination histories of cases born overseas are particularly difficult to verify. Improved recording of clinical details of cases during times of outbreak could improve our understanding of measles infectiousness and better inform our outbreak response and control efforts.

As more countries progress towards measles elimination, transmission in health-care facilities assumes increasing importance as a remaining obstacle. Though imported measles cases will continue to challenge countries that have achieved elimination status, (*21*) health-care-setting transmissions can be addressed more effectively to ensure that health-care facilities are not contributing to outbreaks. Describing characteristics of health-care-setting outbreaks such as this one may assist in improving appropriate and targeted response and control efforts.
